# (*E*)-4-Hydr­oxy-*N*′-(4-nitro­benzyl­idene)benzohydrazide

**DOI:** 10.1107/S1600536809020066

**Published:** 2009-06-06

**Authors:** Cong-Ming Li, Hong-Yan Ban

**Affiliations:** a College of Science, Shenyang University, Shenyang 110044, People’s Republic of China; b School of Chemical Engineering, University of Science and Technology Liaoning, Anshan 114051, People’s Republic of China

## Abstract

The mol­ecule of the title compound, C_14_H_11_N_3_O_4_, is approximately planar, the dihedral angle between the planes of the two substituted benzene rings being 2.54 (7)°. The mol­ecule exists in a *trans* configuration with respect to the central methyl­idene unit. In the crystal structure, mol­ecules are linked through inter­molecular O—H⋯O, N—H⋯O and C—H⋯O hydrogen bonds, forming layers parallel to (101). The O/N—H⋯O and C—H⋯O inter­actions form a pair of bifurcated acceptor bonds involving the cabon­yl/nitro O atom, generating an *R*_2_^1^(6) motif.

## Related literature

For the biological activity of hydrazones, see: Zhong *et al.* (2007 ▶); Raj *et al.* (2007 ▶); Jimenez-Pulido *et al.* (2008 ▶). For related structures, see: Ban & Li (2008*a* ▶,*b* ▶); Li & Ban (2009*a* ▶,*b* ▶); Yehye *et al.* (2008 ▶); Fun *et al.* (2008*a* ▶,*b* ▶); Yang *et al.* (2008 ▶); Ejsmont *et al.* (2008 ▶).
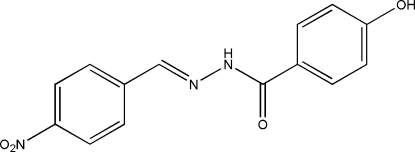


## Experimental

### 

#### Crystal data

C_14_H_11_N_3_O_4_
*M* *_r_* = 285.26
Monoclinic, 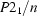

*a* = 7.659 (1) Å
*b* = 13.587 (2) Å
*c* = 12.561 (2) Å
β = 92.784 (5)°
*V* = 1305.6 (3) Å^3^
*Z* = 4
Mo *K*α radiation
μ = 0.11 mm^−1^
*T* = 298 K
0.20 × 0.20 × 0.18 mm

#### Data collection

Bruker SMART CCD area-detector diffractometer
Absorption correction: multi-scan (*SADABS*; Sheldrick, 1996 ▶) *T*_min_ = 0.979, *T*_max_ = 0.981
7862 measured reflections
2835 independent reflections
2109 reflections with *I* > 2σ(*I*)
*R*_int_ = 0.027

#### Refinement

*R*[*F* ^2^ > 2σ(*F* ^2^)] = 0.040
*wR*(*F* ^2^) = 0.111
*S* = 1.04
2835 reflections
195 parameters
1 restraint
H atoms treated by a mixture of independent and constrained refinement
Δρ_max_ = 0.21 e Å^−3^
Δρ_min_ = −0.13 e Å^−3^

### 

Data collection: *SMART* (Bruker, 1998 ▶); cell refinement: *SAINT* (Bruker, 1998 ▶); data reduction: *SAINT*; program(s) used to solve structure: *SHELXS97* (Sheldrick, 2008 ▶); program(s) used to refine structure: *SHELXL97* (Sheldrick, 2008 ▶); molecular graphics: *SHELXTL* (Sheldrick, 2008 ▶); software used to prepare material for publication: *SHELXTL*.

## Supplementary Material

Crystal structure: contains datablocks global, I. DOI: 10.1107/S1600536809020066/ci2812sup1.cif

Structure factors: contains datablocks I. DOI: 10.1107/S1600536809020066/ci2812Isup2.hkl

Additional supplementary materials:  crystallographic information; 3D view; checkCIF report

## Figures and Tables

**Table 1:** Hydrogen-bond geometry (Å, °)

*D*—H⋯*A*	*D*—H	H⋯*A*	*D*⋯*A*	*D*—H⋯*A*
O2—H2⋯O1^i^	0.82	2.02	2.8131 (15)	164
N2—H2*B*⋯O4^ii^	0.90 (1)	2.22 (1)	3.0513 (17)	155 (2)
C7—H7⋯O4^ii^	0.93	2.41	3.235 (2)	148
C13—H13⋯O1^i^	0.93	2.35	3.0713 (19)	134

Symmetry codes: (i) 
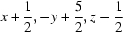
; (ii) 
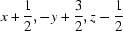
.

**Fig. 1.**
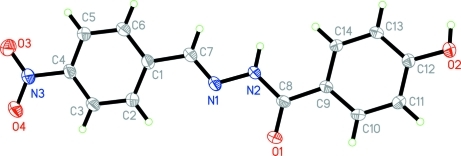
The molecular structure of the title compound, showing 30% probability displacement ellipsoids for the non-hydrogen atoms.

**Fig. 2.**
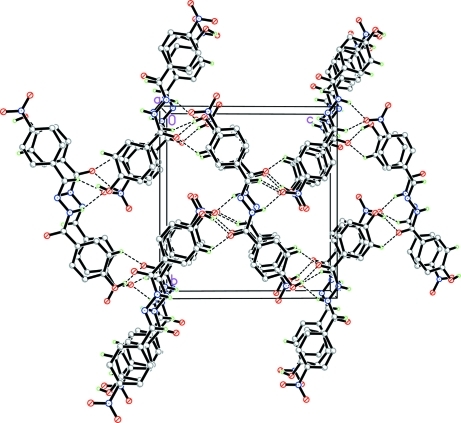
The packing diagram of the title compound, viewed along the a axis. Hydrogen bonds are shown as dashed lines.

## supplementary crystallographic information

### Comment

Schiff bases derived from the condensation of aldehydes with hydrazides have been demonstrated to possess excellent biological activities (Zhong *et al.*, 2007; Raj *et al.*, 2007; Jimenez-Pulido *et al.*, 2008). Due to the easy synthesis of such compounds, a great deal of Schiff bases have been synthesized and structurally characterized (Yehye *et al.*, 2008; Fun *et al.*, 2008a,b; Yang *et al.*, 2008; Ejsmont *et al.*, 2008). Recently, we have reported a few such compounds (Ban & Li, 2008a,b; Li & Ban, 2009a,b). We report here the crystal structure of the title new compound.

The title Schiff base molecule (Fig. 1) is nearly planar, with the dihedral angle between the two benzene rings being 2.54 (7)°. The molecule exists in a *trans* configuration with respect to the central methylidene (C7═N1) unit. The N2—N1—C7—C1 torsion angle is 179.12 (14)°.

In the crystal structure, the molecules are linked through intermolecular O—H···O and N—H···O hydrogen bonds (Table 1), forming layers parallel to the (101) (Fig. 2). Within the layer, C—H···O hydrogen bonds are also observed.

### Experimental

The title compound was prepared by refluxing 4-nitrobenzaldehyde (1.0 mol) with 4-hydroxybenzohydrazide (1.0 mol) in methanol (100 ml). Excess methanol was removed from the mixture by distillation. A colourless solid product was filtered, and washed three times with methanol. Colourless block-shaped crystals of the title compound were obtained from a methanol solution by slow evaporation in air.

### Refinement

Atom H2B was located in a difference Fourier map and refined isotropically, with the N–H distance restrained to 0.90 (1)Å and U_iso_ fixed at 0.08 Å^2^. The remaining H atoms were placed in calculated positions (C–H = 0.93 Å and O–H = 0.82 Å) and refined as riding with U_iso_(H) = 1.2U_eq_(C) and 1.5U_eq_(O).

### Crystal data

**Table:**

C_14_H_11_N_3_O_4_	*F*(000) = 592
*M**_r_* = 285.26	*D*_x_ = 1.451 Mg m^−^^3^
Monoclinic, *P*2_1_/*n*	Mo *K*α radiation, λ = 0.71073 Å
Hall symbol: -P 2yn	Cell parameters from 2390 reflections
*a* = 7.659 (1) Å	θ = 3.0–30.5°
*b* = 13.587 (2) Å	µ = 0.11 mm^−^^1^
*c* = 12.561 (2) Å	*T* = 298 K
β = 92.784 (5)°	Block, colourless
*V* = 1305.6 (3) Å^3^	0.20 × 0.20 × 0.18 mm
*Z* = 4	

### Data collection

**Table:**

Bruker SMART CCD area-detector diffractometer	2835 independent reflections
Radiation source: fine-focus sealed tube	2109 reflections with *I* > 2σ(*I*)
graphite	*R*_int_ = 0.027
ω scans	θ_max_ = 27.0°, θ_min_ = 2.2°
Absorption correction: multi-scan (*SADABS*; Sheldrick, 1996)	*h* = −9→9
*T*_min_ = 0.979, *T*_max_ = 0.981	*k* = −17→15
7862 measured reflections	*l* = −15→15

### Refinement

**Table:**

Refinement on *F*^2^	Secondary atom site location: difference Fourier map
Least-squares matrix: full	Hydrogen site location: inferred from neighbouring sites
*R*[*F*^2^ > 2σ(*F*^2^)] = 0.040	H atoms treated by a mixture of independent and constrained refinement
*wR*(*F*^2^) = 0.111	*w* = 1/[σ^2^(*F*_o_^2^) + (0.0467*P*)^2^ + 0.3196*P*] where *P* = (*F*_o_^2^ + 2*F*_c_^2^)/3
*S* = 1.03	(Δ/σ)_max_ = 0.001
2835 reflections	Δρ_max_ = 0.21 e Å^−^^3^
195 parameters	Δρ_min_ = −0.13 e Å^−^^3^
1 restraint	Extinction correction: *SHELXL97* (Sheldrick, 2008), Fc^*^=kFc[1+0.001xFc^2^λ^3^/sin(2θ)]^-1/4^
Primary atom site location: structure-invariant direct methods	Extinction coefficient: 0.0104 (15)

### Special details

**Table:**

Geometry. All esds (except the esd in the dihedral angle between two l.s. planes) are estimated using the full covariance matrix. The cell esds are taken into account individually in the estimation of esds in distances, angles and torsion angles; correlations between esds in cell parameters are only used when they are defined by crystal symmetry. An approximate (isotropic) treatment of cell esds is used for estimating esds involving l.s. planes.
Refinement. Refinement of F^2^ against ALL reflections. The weighted R-factor wR and goodness of fit S are based on F^2^, conventional R-factors R are based on F, with F set to zero for negative F^2^. The threshold expression of F^2^ > 2sigma(F^2^) is used only for calculating R-factors(gt) etc. and is not relevant to the choice of reflections for refinement. R-factors based on F^2^ are statistically about twice as large as those based on F, and R- factors based on ALL data will be even larger.

### Fractional atomic coordinates and isotropic or equivalent isotropic displacement parameters (Å^2^)

**Table:**

	*x*	*y*	*z*	*U*_iso_*/*U*_eq_	
N1	0.20673 (17)	0.97706 (9)	1.04460 (10)	0.0410 (3)	
N2	0.26363 (19)	1.05171 (9)	0.98149 (10)	0.0431 (3)	
N3	0.0157 (2)	0.54652 (10)	1.21921 (11)	0.0504 (4)	
O1	0.18428 (15)	1.16731 (8)	1.09824 (8)	0.0472 (3)	
O2	0.51173 (18)	1.44263 (8)	0.75115 (9)	0.0541 (3)	
H2	0.5548	1.4194	0.6982	0.081*	
O3	0.0321 (2)	0.46801 (10)	1.17422 (12)	0.0883 (6)	
O4	−0.04623 (18)	0.55386 (9)	1.30667 (10)	0.0611 (4)	
C1	0.1672 (2)	0.80400 (11)	1.06387 (12)	0.0391 (4)	
C2	0.1011 (2)	0.80979 (11)	1.16524 (12)	0.0423 (4)	
H2A	0.0898	0.8707	1.1980	0.051*	
C3	0.0527 (2)	0.72557 (12)	1.21665 (12)	0.0418 (4)	
H3	0.0085	0.7288	1.2842	0.050*	
C4	0.0707 (2)	0.63608 (11)	1.16619 (12)	0.0402 (4)	
C5	0.1366 (2)	0.62796 (12)	1.06648 (13)	0.0455 (4)	
H5	0.1483	0.5668	1.0344	0.055*	
C6	0.1845 (2)	0.71259 (12)	1.01580 (12)	0.0450 (4)	
H6	0.2290	0.7087	0.9484	0.054*	
C7	0.2198 (2)	0.89103 (11)	1.00586 (13)	0.0439 (4)	
H7	0.2642	0.8835	0.9387	0.053*	
C8	0.25098 (19)	1.14649 (11)	1.01458 (11)	0.0354 (3)	
C9	0.32220 (19)	1.22217 (11)	0.94326 (11)	0.0348 (3)	
C10	0.3187 (2)	1.32057 (11)	0.97329 (12)	0.0404 (4)	
H10	0.2734	1.3378	1.0381	0.048*	
C11	0.3812 (2)	1.39297 (11)	0.90850 (13)	0.0447 (4)	
H11	0.3772	1.4585	0.9298	0.054*	
C12	0.4503 (2)	1.36889 (11)	0.81144 (12)	0.0390 (4)	
C13	0.4543 (2)	1.27096 (11)	0.78040 (12)	0.0410 (4)	
H13	0.4995	1.2538	0.7156	0.049*	
C14	0.3915 (2)	1.19946 (11)	0.84559 (12)	0.0403 (4)	
H14	0.3952	1.1340	0.8240	0.048*	
H2B	0.303 (3)	1.0358 (15)	0.9176 (10)	0.080*	

### Atomic displacement parameters (Å^2^)

**Table:**

	*U*^11^	*U*^22^	*U*^33^	*U*^12^	*U*^13^	*U*^23^
N1	0.0486 (8)	0.0377 (7)	0.0377 (7)	−0.0014 (6)	0.0127 (6)	0.0081 (6)
N2	0.0616 (9)	0.0335 (7)	0.0359 (7)	−0.0021 (6)	0.0200 (6)	0.0028 (5)
N3	0.0650 (10)	0.0397 (8)	0.0477 (8)	−0.0055 (7)	0.0146 (7)	0.0081 (6)
O1	0.0630 (7)	0.0441 (6)	0.0364 (6)	0.0005 (5)	0.0217 (5)	−0.0009 (5)
O2	0.0760 (9)	0.0361 (6)	0.0525 (7)	−0.0056 (6)	0.0280 (6)	0.0047 (5)
O3	0.1579 (16)	0.0359 (7)	0.0749 (10)	−0.0156 (9)	0.0465 (10)	−0.0013 (7)
O4	0.0814 (9)	0.0505 (7)	0.0537 (7)	−0.0059 (6)	0.0289 (7)	0.0118 (6)
C1	0.0401 (8)	0.0381 (8)	0.0395 (8)	−0.0015 (6)	0.0068 (6)	0.0070 (6)
C2	0.0511 (10)	0.0350 (8)	0.0413 (9)	−0.0008 (7)	0.0087 (7)	0.0012 (6)
C3	0.0477 (9)	0.0425 (9)	0.0358 (8)	0.0001 (7)	0.0103 (7)	0.0051 (7)
C4	0.0440 (9)	0.0364 (8)	0.0408 (8)	−0.0029 (7)	0.0073 (7)	0.0076 (6)
C5	0.0563 (10)	0.0368 (8)	0.0446 (9)	−0.0019 (7)	0.0129 (8)	0.0002 (7)
C6	0.0546 (10)	0.0436 (9)	0.0379 (8)	−0.0008 (7)	0.0141 (7)	0.0029 (7)
C7	0.0526 (10)	0.0418 (9)	0.0384 (8)	−0.0023 (7)	0.0135 (7)	0.0047 (7)
C8	0.0375 (8)	0.0388 (8)	0.0307 (7)	0.0026 (6)	0.0091 (6)	0.0001 (6)
C9	0.0378 (8)	0.0342 (7)	0.0331 (7)	0.0017 (6)	0.0095 (6)	−0.0003 (6)
C10	0.0495 (9)	0.0379 (8)	0.0352 (8)	0.0012 (7)	0.0158 (7)	−0.0050 (6)
C11	0.0568 (10)	0.0313 (8)	0.0473 (9)	−0.0003 (7)	0.0161 (7)	−0.0051 (7)
C12	0.0432 (9)	0.0333 (7)	0.0414 (8)	−0.0014 (6)	0.0111 (7)	0.0027 (6)
C13	0.0516 (9)	0.0385 (8)	0.0345 (8)	−0.0003 (7)	0.0178 (7)	−0.0022 (6)
C14	0.0529 (9)	0.0302 (7)	0.0394 (8)	−0.0006 (6)	0.0165 (7)	−0.0034 (6)

### Geometric parameters (Å, °)

**Table:**

N1—C7	1.272 (2)	C4—C5	1.377 (2)
N1—N2	1.3714 (17)	C5—C6	1.373 (2)
N2—C8	1.3581 (19)	C5—H5	0.93
N2—H2B	0.899 (9)	C6—H6	0.93
N3—O3	1.2164 (18)	C7—H7	0.93
N3—O4	1.2219 (17)	C8—C9	1.4848 (19)
N3—C4	1.4590 (19)	C9—C10	1.390 (2)
O1—C8	1.2237 (16)	C9—C14	1.395 (2)
O2—C12	1.3539 (17)	C10—C11	1.377 (2)
O2—H2	0.82	C10—H10	0.93
C1—C6	1.390 (2)	C11—C12	1.392 (2)
C1—C2	1.395 (2)	C11—H11	0.93
C1—C7	1.456 (2)	C12—C13	1.387 (2)
C2—C3	1.374 (2)	C13—C14	1.373 (2)
C2—H2A	0.93	C13—H13	0.93
C3—C4	1.381 (2)	C14—H14	0.93
C3—H3	0.93		
			
C7—N1—N2	115.18 (12)	C1—C6—H6	119.5
C8—N2—N1	119.67 (12)	N1—C7—C1	121.73 (14)
C8—N2—H2B	122.3 (14)	N1—C7—H7	119.1
N1—N2—H2B	117.9 (14)	C1—C7—H7	119.1
O3—N3—O4	122.86 (14)	O1—C8—N2	121.40 (13)
O3—N3—C4	118.74 (14)	O1—C8—C9	122.61 (13)
O4—N3—C4	118.40 (14)	N2—C8—C9	115.99 (12)
C12—O2—H2	109.5	C10—C9—C14	117.70 (13)
C6—C1—C2	119.55 (14)	C10—C9—C8	119.34 (13)
C6—C1—C7	118.28 (14)	C14—C9—C8	122.96 (13)
C2—C1—C7	122.18 (14)	C11—C10—C9	120.99 (14)
C3—C2—C1	120.02 (14)	C11—C10—H10	119.5
C3—C2—H2A	120.0	C9—C10—H10	119.5
C1—C2—H2A	120.0	C10—C11—C12	120.51 (14)
C2—C3—C4	118.84 (14)	C10—C11—H11	119.7
C2—C3—H3	120.6	C12—C11—H11	119.7
C4—C3—H3	120.6	O2—C12—C13	122.66 (13)
C5—C4—C3	122.48 (14)	O2—C12—C11	118.25 (13)
C5—C4—N3	118.35 (14)	C13—C12—C11	119.09 (14)
C3—C4—N3	119.16 (13)	C14—C13—C12	119.87 (13)
C6—C5—C4	118.20 (15)	C14—C13—H13	120.1
C6—C5—H5	120.9	C12—C13—H13	120.1
C4—C5—H5	120.9	C13—C14—C9	121.84 (14)
C5—C6—C1	120.91 (14)	C13—C14—H14	119.1
C5—C6—H6	119.5	C9—C14—H14	119.1

### Hydrogen-bond geometry (Å, °)

**Table:**

*D*—H···*A*	*D*—H	H···*A*	*D*···*A*	*D*—H···*A*
O2—H2···O1^i^	0.82	2.02	2.8131 (15)	164
N2—H2B···O4^ii^	0.90 (1)	2.22 (1)	3.0513 (17)	155 (2)
C7—H7···O4^ii^	0.93	2.41	3.235 (2)	148
C13—H13···O1^i^	0.93	2.35	3.0713 (19)	134

Symmetry codes: (i) *x*+1/2, −*y*+5/2, *z*−1/2; (ii) *x*+1/2, −*y*+3/2, *z*−1/2.
